# Timing of chemotherapy‐induced neutropenia is a prognostic factor in patients with advanced gastric cancer undergoing first‐line chemotherapy with oxaliplatin and capecitabine: a retrospective study

**DOI:** 10.1002/cam4.1308

**Published:** 2018-03-13

**Authors:** Yanrong Wang, Yang Chen, Hongyan Yin, Xiaobin Gu, Yan Shi, Guanghai Dai

**Affiliations:** ^1^ Medical Oncology Department 2 Chinese People's Liberation Army General Hospital Beijing 100853 China; ^2^ Department of Radiation Oncology First Hospital of Peking University Beijing 100034 China

**Keywords:** Advanced gastric cancer, timing, chemotherapy‐induced neutropenia, XELOX chemotherapy, overall survival

## Abstract

Chemotherapy‐induced neutropenia (CIN) has been shown to be associated with improved clinical outcomes in patients with various solid tumors. This study retrospectively assessed the association between timing of CIN and prognosis in 321 patients with advanced gastric cancer (AGC) who finished at least one cycle of chemotherapy with oxaliplatin and capecitabine (XELOX). Primary landmark analyses were restricted to 274 patients who received four cycles of chemotherapy and lived for more than 4 months. CIN was categorized as early‐onset and non‐early‐onset. The correlation between timing of CIN with survival was analyzed by the Kaplan‐Meier method and a Cox proportional hazards model. Relative to patients with non‐early‐onset CIN, those with early‐onset CIN had significantly longer times to disease progression (hazard ratio [HR] 0.574; 95% confidence interval [CI] 0.453–0.729, *P* < 0.001) and death (HR: 0.607; 95% CI: 0.478–0.770, *P* < 0.001), consistent with results from the landmark group. In conclusion, timing of CIN may be a potential prognostic biomarker in patients with AGC receiving first‐line chemotherapy with XELOX. Early‐onset CIN predicts better survival.

## Introduction

Gastric cancer is the third most common cause of cancer‐related deaths worldwide 1. Despite recent advances in recent treatment, the median overall survival (OS) of patients with advanced gastric cancer (AGC) rarely exceeds 1 year, and the 5‐year survival rate is less than 10% 2. Unfortunately, approximately 70% of patients diagnosed with gastric carcinoma are not candidates for surgery 1. Compared with best supportive care alone, chemotherapy has resulted in better survival outcomes, fewer symptoms, and improved quality of life in patients with inoperable gastric cancer 3.

Patients may experience varying types and levels of toxicity during chemotherapy, with chemotherapy‐induced neutropenia (CIN) being one of the most important dose‐limiting toxicities of cytotoxic drugs. However, CIN may predict a better prognosis. For example, an analysis of 1265 patients with non‐small‐cell‐lung‐cancer (NSCLC) who received chemotherapy found that neutropenia during chemotherapy was associated with longer survival and lack of neutropenia may indicate underdosing 4. Both mild (grade 1‐2) and severe (grade 3‐4) CIN were found to be favorable prognostic factors to almost the same degree 5. Similarly, a study in 1055 AGC patients treated with S‐1 (oral fluoropyrimidine) found that prognosis was similar in patients with mild (grade 1), moderate (grade 2), and severe (grade 3‐4) neutropenia 6. In patients with small‐cell‐lung‐cancer (SCLC), however, grade 3 or 4 CIN was prognostic of improved OS 7. Despite these findings, these studies were not consistent in determining the relationship between the degree of CIN and clinical outcomes. Other novel molecular biomarkers are associated with high costs, time‐consuming laboratory experiments. Therefore, another easily measurable chemotherapy prognostic marker that can be used for predicting prognosis is desirable.

More recently, the timing (onset) of CIN was found to be predictive of survival in patients with metastatic NSCLC who were treated with first‐line gemcitabine‐platinum doublet chemotherapy 8. However, the prognostic role of timing of CIN has not been assessed in patients with AGC. This study therefore investigated the association between timing of CIN and prognosis in patients with AGC.

## Patients and Methods

### Patients

Between January 1, 2009 and December 31, 2014, 596 patients admitted to Chinese People's Liberation Army (PLA) General Hospital were diagnosed with AGC. Of these, 321 patients who completed at least one cycle of first‐line XELOX chemotherapy were eligible. Patients were included if they (1) were diagnosed with AGC, as confirmed histologically and radiographically; (2) were ineligible for surgery; (3) were <75 years of age; (4) had an Eastern Cooperative Oncology Group (ECOG) performance status (PS) of 0 or 1; (5) had sufficient bone marrow function (neutrophils ≥ 2.0 × 10^9^/L, platelets ≥ 100 × 10^9^/L, hemoglobin ≥ 9.0 g/dL); (6) had normal liver and renal function; (7) had no history of chemotherapy before the commencement of XELOX treatment; (8) did not have bone marrow metastasis; and (9) had no history of radiotherapy. Patients were excluded if (1) they were lost to follow‐up; or (2) had incomplete records of adverse effects of chemotherapy.

Written informed consent was obtained from the patients or their legal guardians before chemotherapy. The study protocol was approved by the Ethics Committee of Chinese People's Liberation Army (PLA) General Hospital, with all aspects of the study complying with the Declaration of Helsinki.

**Figure 1:**
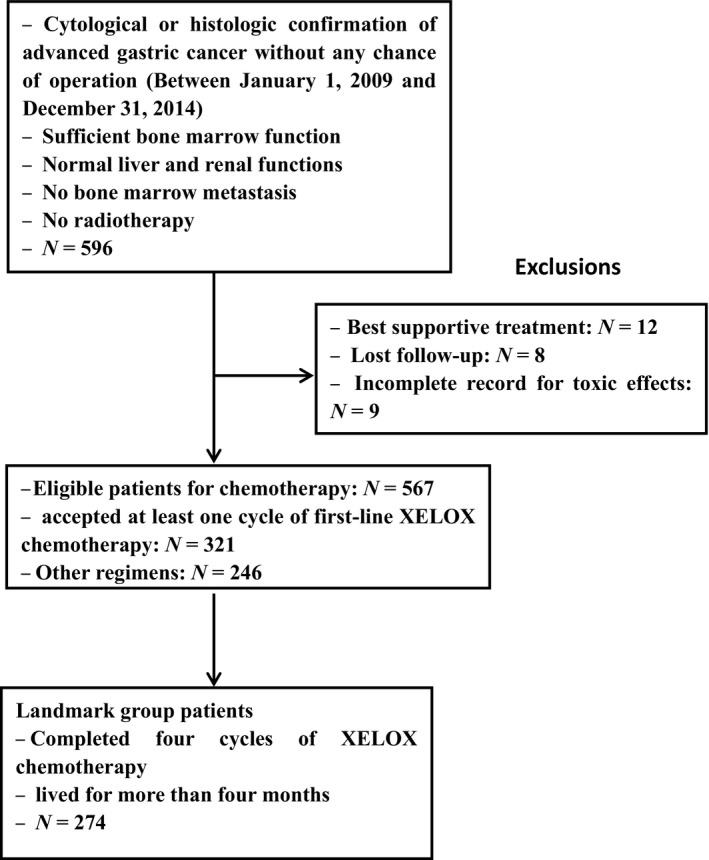
Study flowchart.

Medical information was retrospectively collected from the medical records of PLA General Hospital Registry. The patients were followed up until September 1, 2017 to obtain survival information. Specific details of enrollment and exclusion are shown in Figure 1.

### Treatment

All patients received at least one cycle of XELOX chemotherapy, consisting of an intravenous 2 h infusion of oxaliplatin (130 mg/m^2^) on day 1, and oral capecitabine of (1000 mg/m^2^ twice daily) on days 1–14, every 21 days.

Patients were administered granulocyte colony‐stimulating factor (G‐CSF) for grade 4 neutropenia or febrile neutropenia, but prophylactic G‐CSF was not allowed. The dose of oxaliplatin or capecitabine was reduced by 20% in patients with grade 3 neutropenia. Chemotherapy was delayed in all patients with grade 4 or febrile neutropenia until neutrophil count exceeded 1.5 × 10^9^/L.

### Relative dose intensity of chemotherapy

Relative dose intensity (RDI) of every drug was defined as the ratio between the delivered dose intensity and the planned dose intensity per unit of time, as indicated by the protocol. The RDI mentioned in this study was the mean RDI of oxaliplatin and capecitabine. A dose reduction was defined <90% of the initial planned dose 9.

### Assessment of neutropenia

Routine blood samples were taken before treatment and at the end of every 21‐day chemotherapy cycle (i.e., 1 week after the last dose of oral capecitabine). The worst grade of neutropenia was defined as the lowest neutrophil count recorded between day 1 of the first chemotherapy cycle and 3 weeks after the last cycle. Absolute neutrophil counts (ANCs) were computed by multiplying the white blood cell count by the total percentage of neutrophils. ANC grades were determined by National Cancer Institute (NCI) Common Terminology Criteria for Adverse Events (CTCAE, version 3.0), with Grades 1–4 defined as 1.5–2.0 × 10^9^/L, 1.0–1.5 × 10^9^/L, 0.5–1.0 × 10^9^/L, and <0.5 × 10^9^/L, respectively 10. Timing of CIN was categorized as early‐onset if the lowest grade of neutropenia (ANC <2.0 × 10^9^/L) occurred during cycle 1 or 2 and as non‐early‐onset neutropenia if patients did not experience ANC< 2.0 × 10^9^/L or if it occurred after cycle 2 8, 11.

### Statistical analysis

Landmark analysis 12 has been used extensively to compare time‐to‐event outcomes during study follow‐up 13. In this study, 321 AGC patients finished at least one cycle of XELOX chemotherapy, with only 274 completing four cycles and living for more than 4 months. The cutoff time was defined as 120 days after the commencement of chemotherapy to avoid selection bias resulting from the inherently better prognosis due to a higher probability of neutropenia as the number of chemotherapy cycles increased 4.

The primary study endpoint was overall survival (OS), defined as the interval between the day of starting XELOX treatment and the date of death or last follow‐up. The secondary study endpoint was progression‐free survival (PFS), was defined as the interval between the day of initiating chemotherapy and the date of first evidence of disease progression or death due to any cause.

The baseline characteristics of patients were summarized using descriptive statistics and intergroup parameters were compared using Pearson's chi‐square tests. Survival curves for PFS and OS were generated by the Kaplan–Meier method and compared by log‐rank tests. Univariate and multivariate analysis of factors associated with OS and PFS was assessed using a Cox proportional hazards model. All statistical analyses were performed using SPSS software (version 19.0; SPSS, Chicago, IL), with a *P* value < 0.05 considered statistically significant. In COX model, we adjusted for gender; age; ECOG PS; baseline hemoglobin concentration; baseline neutrophil, lymphocyte, and platelet counts; differentiation; liver and peritoneal metastases; number of metastatic sites; and timing of CIN.

## Results

### Baseline characteristics

Between January 1, 2009 and December 31, 2014, 321 patients with histologically confirmed AGC received at least one cycle of XELOX chemotherapy, with 274 completing four cycles and living for more than 4 months (Fig.**** 1). This study used a cutoff time of 120 days after the commencement of chemotherapy to avoid selection bias resulting from an inherently better prognosis due to a higher probability of neutropenia with increasing cycles of chemotherapy. Baseline demographic and clinical characteristics were well balanced in all patients and landmark group patients with early‐onset and non‐early‐onset CIN (Table 1).

**Table 1:** Patient characteristics by timing of CIN during first‐line chemotherapy in all patients and landmark group patients

	All patients		Landmark patients	
	Early‐onset	Non‐early‐onset	Early‐onset	Non‐early‐onset
	*N* = 191 (60%)	*N* = 130 (40%)	*N* = 170 (60%)	*N* = 104 (40%)
Gender				
Male	150 (79)	99 (76)	133 (78)	80 (77)
Female	41 (21)	31 (24)	37 (22)	24 (23)
Age, year				
≤57	98 (51)	65 (50)	89 (52)	51 (49)
>57	93 (49)	65 (50)	81 (48)	53 (51)
ECOG PS				
0–1	162 (85)	105 (81)	144 (85)	88 (85)
2	29 (15)	25 (19)	26 (15)	16 (15)
Baseline hemoglobin, g/dL	120.24 ± 20.297	118.77 ± 23.516	120.99 ± 20.221	120.41 ± 23.240
Baseline neutrophils, × 10^9^/L	4.10 ± 1.624	4.64 ± 2.060	4.05 ± 1.531	4.49 ± 1.906
Baseline lymphocytes, × 10^9^/L	1.56 ± 0.525	1.58 ± 0.716	1.57 ± 0.518	1.55 ± 0.570
Baseline platelets, × 10^9^/L	246.68 ± 84.602	256.78 ± 89.474	247.13 ± 86.862	246.12 ± 83.883
Differentiation				
Well‐moderate	54 (28)	38 (29)	52 (31)	32 (31)
Poor	137 (72)	92 (71)	118 (69)	72 (69)
Liver metastasis				
Absent	85 (45)	51 (39)	78 (46)	40 (39)
Present	106 (55)	79 (61)	92 (54)	64 (61)
Peritoneal metastasis				
Absent	141 (74)	85 (65)	122 (72)	69 (66)
Present	50 (26)	45 (35)	48 (28)	35 (34)
Number of metastatic sites				
≤2	159 (83)	90 (69)	144 (85)	73 (70)
>2	32 (17)	40 (31)	26 (15)	31 (30)
Degree of CIN				
0	55 (29)	40 (31)	48 (28)	24 (26)
1–2	93 (49)	64 (49)	83 (49)	56 (51)
3–4	43 (22)	26 (20)	39 (23)	24 (23)

ECOG PS, Eastern Cooperative Oncology Group Performance Status; RDI, relative dose intensity; CIN, chemotherapy‐induced neutropenia.

### Assessment of neutropenia

Of the 321 patients, 192 (60%) experienced early‐onset and 130 (40%) experienced non‐early‐onset CIN. Of the 274 landmark group patients, 170 (62%) experienced early‐onset neutropenia and 104 (38%) experienced non‐early‐onset neutropenia.

### Survival analysis in all patients and landmark group patients

**Figure 2:**
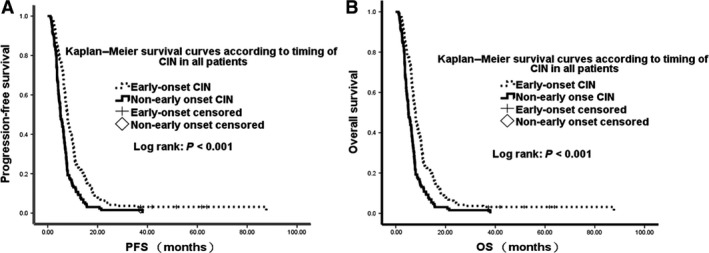
Kaplan–Meier analysis of progression‐free survival and overall survival by timing of neutropenia in the full patient cohort.

**Figure 3:**
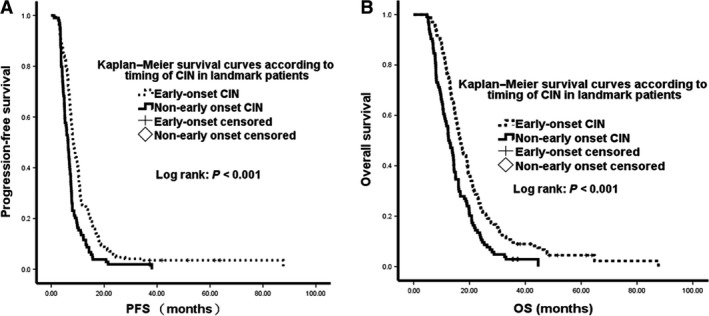
Kaplan–Meier analysis of progression‐free survival and overall survival by timing of neutropenia in the landmark group of patients.

The median follow‐up time for all patients was 32 months (range, 4–78 months). These patients had a median PFS of 6.9 months (95% confidence interval [CI] 6.3–7.4 months) and a median OS of 14.2 months (95% CI: 13.0–15.1 months). In the landmark group, the median PFS was 7.5 months (95% CI: 7.0–7.9 months) and the median OS was 15.0 months (95% CI: 14.3–16.2 months). The correlation between timing of CIN and survival was analyzed by the Kaplan–Meier method and a Cox proportional hazards model. Kaplan–Meier survival curves for all patients and for landmark group patients stratified according to timing of CIN are shown in Figures 2 and 3, respectively.

Univariate analysis analyzed the correlation with survival of several variables, including gender; age; ECOG PS; baseline hemoglobin concentration; baseline neutrophil, lymphocyte, and platelet counts; differentiation; liver and peritoneal metastases; number of metastatic sites; and degree and timing of CIN (Table 2). Univariate analysis in all patients showed that median OS (15.7 vs. 11.3 months, *P* < 0.001) and PFS (7.9 vs. 5.1 months, *P* < 0.001) were significantly longer in patients with early‐onset than with non‐early‐onset neutropenia. Similarly, analysis in landmark group patients analysis showed that median OS (16.7 vs. 12.8 months, *P* < 0.001) and PFS (8.3 vs. 6.3 months, *P* < 0.001) were significantly longer in patients with early‐onset than with non‐early‐onset neutropenia. However, the degree of CIN was not significantly associated with survival outcomes, both in all patients and in the landmark group.

**Table 2:** Univariate analysis of the association between clinical characteristics and survival in all patients and landmark group patients diagnosed with advanced gastric cancer

	All patients						Landmark patients					
	PFS			OS			PFS			OS		
	HR	95% CI	*P* value	HR	95% CI	*P* value	HR	95% CI	*P* value	HR	95% CI	*P* value
Gender												
Male	1			1			1			1		
Female	1.105	0.847–1.440	0.462	1.137	0.870–1.485	0.347	1.144	0.857–1.527	0.362	1.171	0.875–1.567	0.289
Age, year												
≤57	1			1			1			1		
>57	1.140	0.913–1.425	0.247	1.109	0.887–1.386	0.364	1.151	0.904–1.465	0.253	1.106	0.868–1.408	0.416
ECOG PS												
0–1	1			1			1			1		
2	1.181	0.877–1.590	0.274	1.436	1.064–1.936	0.018	1.090	0.778–1.525	0.617	1.343	0.958–1.883	0.087
Baseline hemoglobin, g/dL	0.998	0.992–1.003	0.356	0.996	0.991–1.001	0.150	1.000	0.994–1.005	0.880	0.997	0.992–1.003	0.393
Baseline neutrophils, × 10^9^/L	1.014	0.951–1.081	0.677	1.038	0.972–1.108	0.264	0.970	0.903–1.042	0.408	0.995	0.924–1.071	0.889
Baseline lymphocytes, × 10^9^/L	0.943	0.771–1.153	0.565	0.864	0.704–1.062	0.164	0.879	0.703–1.098	0.257	0.812	0.644–1.025	0.079
Baseline platelets, × 10^9^/L	1.002	1.000–1.003	0.009	1.001	1.000–1.003	0.028	1.001	1.000–1.003	0.114	1.001	1.000–1.002	0.181
Differentiation												
Well‐moderate	1			1			1			1		
Poor	1.433	1.120–1.834	0.004	1.713	1.333–2.202	<0.001	1.377	1.060–1.789	0.017	1.715	1.313–2.240	<0.001
Liver metastasis												
Absent	1			1			1			1		
Present	1.032	0.825–1.292	0.781	0.873	0.696–1.096	0.243	0.972	0.763–1.240	0.822	0.813	0.635–1.041	0.101
Peritoneal metastasis												
Absent	1			1			1			1		
Present	1.194	0.936–1.524	0.153	1.136	1.083–1.774	0.01	1.308	1.005–1.701	0.046	1.510	1.156–1.973	0.003
Number of metastatic sites												
≤2	1			1			1			1		
>2	1.254	1.098–1.433	0.001	1.309	1.003–1.708	0.047	1.659	1.235–2.230	0.001	1.318	0.980–1.774	0.048
Degree of CIN												
1–2 versus 0	0.512	0.394–0.665	<0.001	0.476	0.366–0.620	<0.001	0.549	0.410–0.736	<0.001	0.493	0.367–0.662	<0.001
3–4 versus 0	0.382	0.276–0.528	<0.001	0.368	0.266–0.511	<0.001	0.436	0.307–0.618	<0.001	0.403	0.283–0.573	<0.001
1–2 versus 3‐4	1.341	1.003–1.792	0.048	1.293	0.965–1.733	0.085	1.260	0.931–1.706	0.135	1.224	0.902–1.661	0.194
Timing of CIN												
Early‐onset	1			1			1			1		
Non‐early‐onset	1.822	1.449–2.290	<0.001	1.766	1.403–2.223	<0.001	1.768	1.376–2.270	<0.001	1.710	1.329–2.199	<0.001

ECOG, Eastern Cooperative Oncology Group; CIN, chemotherapy‐induced neutropenia; PFS, progression‐free survival; OS, overall survival.

Statistical significance was defined as a two‐sided *P* < 0.05.

Hazard ratios and 95% CI for OS and PFS were estimated with Cox's proportional hazards model.

The results of multivariate analysis, using the same parameters as covariates (excluding grade of neutropenia), are shown in Table**** 3. Differentiation, number of metastatic sites, and timing of CIN were independent predictors of PFS, whereas ECOG PS, differentiation, number of metastatic sites, and timing of CIN were independently predictive of OS. Assessment of all patients showed that, relative to those with non‐early‐onset CIN, those with early‐onset CIN had significantly longer times to disease progression (hazard ratio [HR]: 0.574; 95% CI: 0.453–0.729, *P* < 0.001) and death (HR 0.607; 95% CI: 0.478–0.770, *P* < 0.001). Similar results were observed in the landmark group.

**Table 3:** Multivariate analysis of the association between clinical characteristics and survival in all patients and landmark group patients diagnosed with advanced gastric cancer

	All patients						Landmark patients					
	PFS			OS			PFS			OS		
	HR	95% CI	*P* value	HR	95% CI	*P* value	HR	95% CI	*P* value	HR	95% CI	*P* value
ECOG PS												
0–1	1			1			1			1		
2	1.178	0.854–1.624	0.317	1.474	1.067–2.036	0.019	1.167	0.813–1.674	0.402	1.470	1.022–2.113	0.038
Baseline platelet counts, × 10^9^/L	1.002	1.000–1.003	0.021									
Differentiation												
Well‐moderate	1			1			1			1		
Poor	1.418	1.091–1.842	0.009	1.700	1.307–2.213	<0.001	1.332	1.006–1.764	0.045	1.675	1.264–2.221	<0.001
Number of metastatic sites												
≤2	1			1			1			1		
>2	1.648	1.243–2.185	0.001	1.392	1.053–1.841	0.020	1.762	1.281–2.422	<0.001	1.478	1.081–2.022	0.014
Timing of CIN												
Non‐early‐onset	1			1			1			1		
Early‐onset	0.574	0.453–0.729	<0.001	0.607	0.478–0.770	<0.001	0.584	0.450–0.758	<0.001	0.619	0.477–0.803	<0.001

CI, confidence interval; HR, hazard ratio; PFS, progression‐free survival; OS, overall survival.

Statistical significance was defined as a two‐sided *P* < 0.05.

Hazard ratios and 95% CIs for OS and PFS were estimated with Cox's proportional hazards models.

Adjusted for gender; age; ECOG PS; baseline hemoglobin concentration; baseline neutrophil, lymphocyte, and platelet counts; differentiation; liver and peritoneal metastases; number of metastatic sites; and timing of CIN.

## Discussion

To our knowledge, this study is the first to investigate the association between timing of CIN and prognosis in patients with AGC. Consistent with previous results, we found that the timing of CIN was predictive of survival, with significantly better survival outcomes observed in patients with early‐onset than with non‐early‐onset CIN 5, 8, 11, 14. For example, PFS and OS were found to be significantly better in patients with early‐ than late‐onset neutropenia, although survival times did not differ in patients with late‐onset and absence of neutropenia 8. In addition, early‐onset CIN during perioperative chemotherapy was found to be predictive of better OS and disease‐free survival DFS in patients with completely resected NSCLC 11. Similarly, OS was found to be significantly better in patients with advanced pancreatic cancer receiving gemcitabine/gemcitabine based chemotherapy who experienced early‐onset CIN than those with non‐early‐onset CIN 14.

It was not fully understood why early‐onset neutropenia can predict better clinical outcomes. Although neutropenia is not directly related to better prognosis, many studies, including ours, have suggested that CIN reflects the pharmacokinetics of cytotoxic drugs, the chemosensitivity of antitumor drugs, inflammation in the tumor microenvironment, and/or interactions among some or all of these variables.

Although dose calculations for cytotoxic drugs have been based on body‐surface area (BSA), several reports have suggested that a BSA‐based dosing system may be appropriate or even insufficient or suboptimal in some patients 6, 15. The poor correlation between BSA and the pharmacokinetics of many chemotherapeutic drugs may be caused by variations in patient metabolism 16, 17, 18, 19, 20. Our findings suggest that the early‐onset of CIN may be a surrogate marker for drug metabolism or elimination, which can be used to adjust drug dose appropriately.

The response of cancer cells to chemotherapeutic drugs depends on a sufficient amount of active drug reaching the target and on the sensitivity of the target to the effect of the drug. The availability of an active antitumor drug is affected by pharmacokinetic factors, including the metabolism, distribution, and catabolism of a drug. The chemosensitivity of both tumor cells and healthy cells is affected, in part, by genetic predisposition and interindividual variations in systemic exposure, but is also modified by tumor‐specific acquired resistance 4, 21, 22. Thus, neutrophil counts may act as a surrogate marker of tumor chemosensitivity. The early‐onset of neutropenia indicate neutrophil susceptibility to antitumor drugs and the nonemergence of tumor resistance to that drug 8.

Inflammation in the tumor microenvironment has been found to affect tumor development, invasion, and metastasis 23, 24, 25. Elevated blood neutrophil counts in an inflammatory microenvironment have been shown to contribute to tumor angiogenesis and to induce resistance to antivascular endothelial growth factor therapy, resulting in poor prognosis and response 26, 27, 28, 29, 30. Therefore, a high peripheral neutrophil level may indicate drug resistance and tumor progression, and predict poor prognosis. Therefore, early‐onset CIN may predict better survival because decreased neutrophil counts may slow tumor progression.

Because neutropenia did not occur before the initiation of chemotherapy, the association between neutropenia and prognosis may have been falsely attributed to an association between neutropenia and number of cycles of chemotherapy. The duration of chemotherapy was less likely to affect treatment results, as most patients discontinue following disease progression resulting from tumors being highly malignant and showing invasive biological behavior. Thus, the association between CIN and better outcomes was considered a consequence of selection bias. To avoid this bias, we performed landmark analysis, including patients who had received at least four cycles of XELOX chemotherapy and lived for more than 120 days. Because of the inappropriateness of BSA‐based dosing systems, genetic predisposition may explain the large interindividual variation in systemic exposure.

This study had several limitations. First, it was retrospective in design, with a moderate sample size. Moreover, all patients were Chinese population and received a single chemotherapy regimen, XELOX. Our present findings may therefore be inapplicable to patients treated with other chemotherapeutic regimens. Second, OS as the primary endpoint may have been confounded by different subsequent chemotherapy regimens or radiotherapy. Despite these methodological issues, there is a need for an accurate, easily measurable surrogate marker of patient prognosis and chemosensitivity to antitumor drugs.

In conclusion, this study showed that the timing of CIN may be a potential prognostic biomarker in patients with AGC receiving first‐line XELOX chemotherapy. Early‐onset of CIN may be a surrogate marker for tumor chemosensitivity and optimum dosing of drugs, which could improve clinical outcomes. Well‐designed prospective trials are needed to evaluate the association between CIN and survival in patients with AGC.

## Conflict of Interest

The authors declare no conflict of interests.

## Informed consent

Informed consent was obtained from all individual participants included in the study.
